# Craniosacral therapy for chronic pain: a systematic review and meta-analysis of randomized controlled trials

**DOI:** 10.1186/s12891-019-3017-y

**Published:** 2019-12-31

**Authors:** Heidemarie Haller, Romy Lauche, Tobias Sundberg, Gustav Dobos, Holger Cramer

**Affiliations:** 1 Department of Internal and Integrative Medicine, Evang. Kliniken Essen-Mitte, Faculty of Medicine, University of Duisburg-Essen, Essen, Germany; 2 Department of Internal and Integrative Medicine, Sozialstiftung Bamberg, Bamberg, Germany; 3 Australian Research Centre in Complementary and Integrative Medicine (ARCCIM), Faculty of Health, University of Technology Sydney (UTS), Sydney, Australia; 4 Nursing and Midwifery, Monash University, Melbourne, Australia; 5 Musculoskeletal & Sports Injury Epidemiology Center, Institute of Environmental Medicine, Karolinska Institutet, Stockholm, Sweden

**Keywords:** Chronic pain, Craniosacral therapy, Complementary therapies, Meta-analysis, Systematic review

## Abstract

**Objectives:**

To systematically assess the evidence of Craniosacral Therapy (CST) for the treatment of chronic pain.

**Methods:**

PubMed, Central, Scopus, PsycInfo and Cinahl were searched up to August 2018. Randomized controlled trials (RCTs) assessing the effects of CST in chronic pain patients were eligible. Standardized mean differences (SMD) and 95% confidence intervals (CI) were calculated for pain intensity and functional disability (primary outcomes) using Hedges’ correction for small samples. Secondary outcomes included physical/mental quality of life, global improvement, and safety. Risk of bias was assessed using the Cochrane tool.

**Results:**

Ten RCTs of 681 patients with neck and back pain, migraine, headache, fibromyalgia, epicondylitis, and pelvic girdle pain were included. CST showed greater post intervention effects on: pain intensity (SMD = -0.32, 95%CI = [− 0.61,-0.02]) and disability (SMD = -0.58, 95%CI = [− 0.92,-0.24]) compared to treatment as usual; on pain intensity (SMD = -0.63, 95%CI = [− 0.90,-0.37]) and disability (SMD = -0.54, 95%CI = [− 0.81,-0.28]) compared to manual/non-manual sham; and on pain intensity (SMD = -0.53, 95%CI = [− 0.89,-0.16]) and disability (SMD = -0.58, 95%CI = [− 0.95,-0.21]) compared to active manual treatments. At six months, CST showed greater effects on pain intensity (SMD = -0.59, 95%CI = [− 0.99,-0.19]) and disability (SMD = -0.53, 95%CI = [− 0.87,-0.19]) versus sham. Secondary outcomes were all significantly more improved in CST patients than in other groups, except for six-month mental quality of life versus sham. Sensitivity analyses revealed robust effects of CST against most risk of bias domains. Five of the 10 RCTs reported safety data. No serious adverse events occurred. Minor adverse events were equally distributed between the groups.

**Discussion:**

In patients with chronic pain, this meta-analysis suggests significant and robust effects of CST on pain and function lasting up to six months. More RCTs strictly following CONSORT are needed to further corroborate the effects and safety of CST on chronic pain.

**Protocol registration at Prospero:**

CRD42018111975.

## Background

Chronic pain disorders are the leading global cause of disability and are still increasing in prevalence [1]. Low back and neck pain, headache and migraine considerably affect all age groups from the beginning of adolescence to middle-aged and older adults [1]. The often limited effects and potential side effects of pharmacological treatments for chronic musculoskeletal pain conditions [2] may be reasons why patients frequently use complementary therapies [3–5]. Among them, Craniosacral Therapy (CST) is a typically requested treatment for complaints of the back and neck, headache and migraine, and associated stress-related and mental health problems [6, 7].

Derived from osteopathic manipulative treatment, CST consists of mindful, non-invasive fascial palpation techniques applied between the cranium and sacrum [8, 9]. Besides releasing myofascial structures, CST intends to normalize sympathetic nerve activity, often increased in chronic pain patients, by modifying craniosacral body rhythms [10, 11]. Reducing physiological arousal and switching to the parasympathetic mode [12] has been shown to enhance the body’s ability for physiological regulation and tissue relaxation [13–17], and to decrease chronic pain [18, 19]. While the specific mechanisms of CST are still understudied, clinical trials have shown preliminary evidence for CST on improving patient-reported outcomes, albeit with often unclear risk of bias due to limited methodological study quality [20–22]. To date, RCTs have only been summarized qualitatively [20–24], and no meta-analysis has provided quantitative information about the mean effects of CST.

By conducting a systematic review and meta-analysis, we aimed to pool the existing evidence of CST in pain patients and to assess whether this evidence is robust against the possible risk of systematic bias.

## Materials and methods

This review was conducted in accordance with the Preferred Reporting Items for Systematic Reviews and Meta-Analyses (PRISMA) guidelines [25] and the recommendations of the Cochrane Collaboration [26]. A protocol of the methods used was previously registered at Prospero (CRD42018111975).

### Eligibility criteria

Studies were eligible if they were published as: either full-texts or abstracts of randomized controlled trials (RCTs) or randomized crossover trials that included adult patients with a chronic, non-malignant pain condition of any cause, duration, or intensity. Studies had to examine a type of CST regardless of length or content. Eligible control interventions were active or inactive comparators such as: treatment as usual, waiting list, sham, pharmacological therapies, or non-pharmacological comparators. To be included, studies had to report at least one primary or secondary outcome assessed at the end of the intervention period or at a follow-up point closest to six months after randomization. Pain intensity and functional disability were defined as primary outcomes. Secondary outcomes included physical quality of life, mental quality of life, global improvement, and safety [27]. If a study reported on more than one instrument assessing the same outcome, disease-specific instruments were preferred over generic ones, multi-item over single-item ones, and clinician-rated over patient-rated ones. Safety was operationalized as the number of adverse events (AE) or study withdrawals due to AEs. AEs were defined as any untoward medical occurrence in a patient, which did not have to have a defined causal relationship with the study treatment. Cases of any untoward medical occurrence that, at any dose, has resulted in death, was life-threatening, required inpatient hospitalization, or caused persistent or significant disability were rated as serious AEs [28].

Studies were excluded if they were non-randomized trials, included samples of children or adolescents, or tested interventions that were not defined as CST by the trial authors (for example specific techniques related to cranial osteopathy).

### Literature search

We searched PubMed, PsycInfo, Central Trials, Cinahl, and Scopus from inception to August 2018 by browsing titles, abstracts, and keywords using the search terms “craniosacral” or “cranio sacral”. No language restrictions were applied. We manually searched reference lists of previous studies and reviews, PhD and DO theses, and websites of international craniosacral associations to retrieve additional articles. For ongoing and unpublished studies, we searched international trial registries of the NIC and WHO and conference proceedings. Two reviewers (HH and HC) independently screened titles and abstracts of those studies and assessed the remaining full-texts for eligibility. Any disagreements were rechecked with a third reviewer (RL) until consensus was achieved.

### Data extraction

Two reviewers (RL and HC) independently extracted data from the eligible studies including: their origin, the studied pain condition, the sample size, the mean age of the patients, the percentage of included women, the type, content and lengths of the experimental and control intervention, the outcomes and assessment points included in the meta-analysis, reported AEs, and sources of funding. Discrepancies were rechecked with a third reviewer (HH) until consensus was achieved.

### Risk of bias in individual studies

Again, two reviewers (RL and TS) independently assessed the risk of: selection, performance, detection, attrition, reporting, and other bias using the Cochrane risk of bias tool [26]. Each domain was judged as either, ‘low risk of bias’ if all requirements were adequately fulfilled, ‘high risk of bias’ if the requirements were not adequately fulfilled, and as ‘unclear risk of bias’ if insufficient data for a judgment was provided. Divergent judgments were rechecked with a third reviewer (HC) until consensus was achieved.

### Statistical analyses

#### Assessment of overall effect sizes

Pairwise meta-analyses were conducted by Review Manager Software (RevMan, Version 5.3, The Nordic Cochrane Centre, Copenhagen) using random-effects models (inverse variance method). Effects were pooled for studies comparing CST to treatment as usual or wait list, manual or non-manual sham treatments, active pharmacological treatments, and similar active non-pharmacological treatments at the respective time point. Standardized mean differences (SMDs) with 95% confidence intervals (CIs) were calculated, which indicate the difference in means between groups divided by the pooled standard deviation (SD) using Hedges’ correction for small samples (N) [26]. Where no SDs were available, they were calculated from standard errors, CIs or t-values [26], or were requested from trial authors by email. For pain intensity and functional disability, a negative SMD indicated greater effects of CST compared to the respective control condition. For the quality of life measures and the global improvement ratings, a positive SMD indicated greater effects of CST compared to control. In accordance with Cohen’s categories, Hedges’ g can be interpreted as: a small effect, in cases of an SMD of 0.2–0.5; as a medium effect in cases of an SMD of 0.5–0.8; and as a large effect in cases of an SMD of > 0.8 [29]. Respective categories were applied for negative SMDs.

#### Subgroup analyses

Subgroup analyses were considered for patients with different pain diagnoses and different types of CST but could not be performed, as there were insufficient studies for those comparisons.

#### Assessment of heterogeneity

Chi^2^ statistics were used to explore statistical heterogeneity between studies, with a *p*-value of ≤ .10 indicating significant heterogeneity. The magnitude of heterogeneity was categorized by the I^2^ with: I^2^ > 25%, I^2^ > 50%, and I^2^ > 75% representing moderate, substantial, and considerable heterogeneity, respectively [26, 30].

#### Sensitivity analyses

Where studies with high or unclear risk of bias were pooled with those of low risk of bias, sensitivity analyses were performed to test the robustness of significant effects. If substantial or considerable statistical heterogeneity was present in a meta-analysis, sensitivity analyses were used to explain them as a possible consequence of clinical heterogeneity in study quality, samples, or intervention characteristics.

#### Risk of bias across studies

We intended to use visual analysis of funnel plots to assess publication bias if more than 10 studies could be included in a single meta-analysis [31].

## Results

### Literature search

**Fig. 1:**
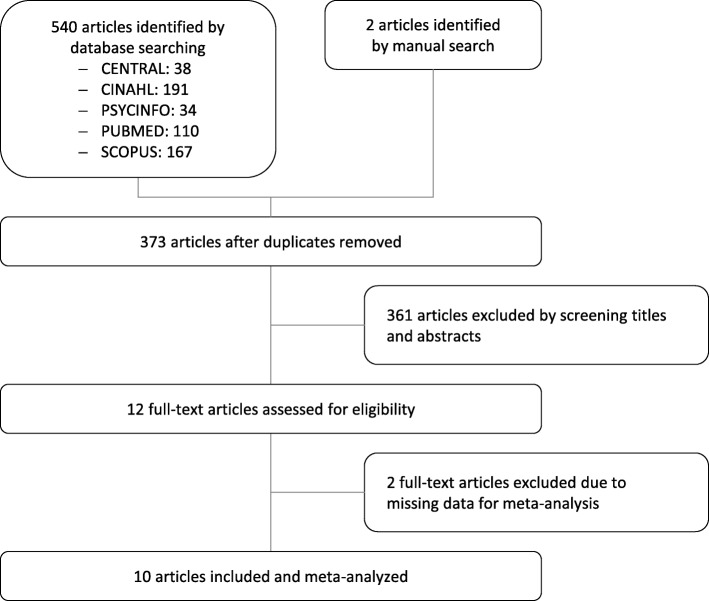
Flowchart of the literature search

The electronic database search revealed 540 articles (Fig. 1). Two additional articles were retrieved from the manual search. After removing duplicates and excluding articles by screening titles and abstracts, 12 full-text articles were assessed for eligibility. Two further articles [32, 33] had to be excluded as they did not report sufficient data for meta-analysis. Another article was only published as a study protocol [34] and a conference proceeding [35] but detailed data was provided by email. Thus, a final sample of 10 RCTs published between 1999 and 2016 that included 681 patients were eligible for meta-analysis [35–44].

### Study characteristics

The characteristics of the included RCTs are presented in Table 1. The RCTs were conducted in: the US [35, 42, 44], Spain [38, 39, 43], Germany [41], Iceland [36], Poland [37], and Sweden [40]. The trials included patients suffering from: tension-type headache [41], migraine [35, 36], low back pain [37, 39], neck pain [41], fibromyalgia [38, 43], pelvic girdle pain [40], and lateral epicondylitis [44]. Sample sizes ranged from 20 to 123 with a median N of 62 and a median of 90% of women. The median age of the total sample was 43.4 years with a range from 30.6 to 52.5 years. Studies provided 1 to 50 CST treatments with a median number of 7 treatments within a maximum of 25 weeks. While two studies used a single CST technique [42, 44], the others implemented a more comprehensive, semi-standardized treatment protocol [35–41, 43]. Control conditions included: treatment as usual [40], no treatment [42], wait list [36], non-manual sham procedures (disconnected devices) [35, 38, 43], manual sham [41, 44], and active manual treatments such as trigger point therapy [37] and soft tissue massage [39]. No study compared CST to an active drug treatment.

**Table 1:** Study characteristics

Reference	Origin	Chronic pain condition	Sample size	Mean age	Percentage of women	Experimental intervention	Control intervention	Assessment points	Outcomes	Safety	Funding
Arnadottir 2013 [36]	Iceland	Migraine	20	37.6 ± 9.3	90%	6 CST treatments (semi-standardized therapy protocol) over 4 weeks	WL	Post	- Functional disability (HIT-6)	N.r.	N.r.
Bialoszewski 2014 [37]	Poland	Low back pain	55	33.0 ± 7.0	N.r.	3 CST treatments (standardized therapy protocol) over 2 weeks	3 trigger point treatments over 2 weeks	Post	- Pain intensity (LPIQ)	N.r.	N.r.
									- Functional disability (LPIQ)		
Castro-Sanchez 2011 [38]	Spain	Fibromyalgia	109	52.5 ± 11.7	100%	40 CST treatments (semi-standardized therapy protocol) over 20 weeks	40 non-manual sham treatments (inactive magnet therapy) over 20 weeks	Post 7mFU	- Global improvement (CGII)	No withdrawal due to AEs	No specific funding
Castro-Sanchez 2016 [39]	Spain	Low back pain	64	50.0 ± 12.0	66%	10 CST treatments (semi-standardized therapy protocol) over 10 weeks	10 soft tissue massage treatments over 10 weeks	Post 3.5mFU	- Pain intensity (NRS)	No withdrawal due to AEs	No specific funding
									- Functional disability (RMQ)		
Elden 2013 [40]	Sweden	Pelvic girdle pain	123	30.6 ± 3.9	100%	5 CST treatments (semi-standardized therapy protocol) over 8 weeks + TAU	TAU	Post	- Pain intensity (VAS)	Patients with minor AEs: CST = 5/63 UC = 6/60	Government research grant
									- Functional disability (ODI)		
									- Physical quality of life (EQ. 5D)		
Haller 2016 [41]	Germany	Neck pain	54	44.6 ± 10.0	81.5%	8 CST treatments (semi-standardized therapy protocol) over 8 weeks	8 sham manual treatments (light touch) over 8 weeks	Post 5mFU	- Pain intensity (VAS)	Patients with minor AEs: CST = 7/27 Sham = 9/27	Funding from university (treatments) and CST associations (publication fee)
									- Functional disability (NDI)		
									- Physical quality of life (SF12-PSC)		
									- Mental quality of life (SF12-MSC)		
									- Global improvement (PGII)		
Hanten 1999 [42]	United States	Tension-type headache	60	36.0 ± 12.0	71.7%	1 CST treatment (single technique)	No treatment	Post	- Pain intensity (VAS)	N.r.	N.r.
Mann 2012 [35]	United States	Migraine	69	42.1 ± 16.0	94.2%	8 CST treatments (semi-standardized therapy protocol) over 8 weeks + TAU	8 non-manual sham treatments (inactive/active magnet therapy) over 8 weeks + TAU	Post	- Pain intensity (Diary: severe headache hours/day)	N.r.	Government research grant
									- Functional disability (MIDAS)		
Mataran-Penarrocha 2011 [43]	Spain	Fibromyalgia	104	49.1 ± 14.1	96.4%	50 CST treatments (semi-standardized therapy protocol) over 25 weeks	50 non-manual sham treatments (disconnected ultrasound) over 25 weeks	Post 11mFU	- Pain intensity (SF36-BP)	Patients with AEs: CST = 0/43 Sham = 0/41	N.r.
									- Functional disability (SF36-PF)		
									- Physical quality of life (SF36-GH)		
									- Mental quality of life (SF36-MH)		
Nourbakhsh 2008 [44]	United States	Lateral Epicondylitis	23	52.4 ± 7.2	39.1%	6 CST treatments (single technique) over 3 weeks	6 manual sham treatments (light touch) over 3 weeks	Post	- Pain intensity (NRS)	N.r.	N.r.
									- Functional disability (PSFS)		

Abbreviations: *AE* Adverse event, *CGII* Clinical Global Impression of Improvement Scale *CST* Craniosacral Therapy, *EQ. 5D* European Quality of Life Measure, *HIT-6* Headache Impact Test, *LPIQ* Laitinen Pain Indicator Questionnaire, *mFU* months of follow-up after randomization, *MIDAS* Migraine Disability Assessment Score, *NDI* Neck Disability Index, N.r.: not reported, *NRS* Numeric Rating Scale, *ODI* Oswestry Disability Index, *PGII* Patient Global Impression of Improvement Scale, *PSFS* Patient Specific Functional Scale, *RMQ* Roland Morris Disability Questionnaire, SF12/36-PCS/MCS/BP/GH/MH/PF: 12/36-Item Short Form Health Survey-Physical Component Score/Mental Component Score/Bodily Pain Subscale/Mental Health Subscale/General Health Subscale/Physical Function Subscale, *TAU* Treatment as usual, *VAS* Visual Analogue Scale, *WL* Waiting list

Pain intensity was mostly measured using Numeric Rating Scales (NRS) and Visual Analogue Scales (VAS) [39–42, 44]. One study reported VAS scores as medians only [40]. However, upon request, trial authors provided means and SDs of the morning and evening pain ratings, which were combined to an average pain score. Two additional studies also assessed VAS/NRS data but did not report related SDs [43] or provided incomplete outcome data comprising of only 72% of the sample [35]. Thus, we had to include alternative measurements taking complete data from the Bodily Pain subscale of the 36-Item Short Form Health Survey (SF-36) and from a pain diary assessing hours of severe headache per day. One study, moreover, used the Intensity of Pain subscale of the Laitinen Pain Indicator Questionnaire (LPIQ) [37]. Functional disability was measured using the Headache Impact Test (HIT-6) [36], the Limitation of Activity subscale of the LPIQ [37], the Roland Morris Disability Questionnaire (RMQ) [39], the Oswestry Disability Index (ODI) [40], the Neck Disability Index (NDI) [41], the Migraine Disability Assessment Score (MIDAS) [35], the Physical Function subscale of the SF-36 [43], and the Patient Specific Functional Scale (PSFS) [44]. Physical and mental quality of life were measured by sub- and component-scores of the SF-12 and SF-36 [41, 43], In addition, one study measured physical quality of life using the European Quality of Life Measure (EQ. 5D) and reported median changes [40]. Upon request, the trial authors provided means and SDs, which led us to calculate an additional meta-analysis although it included only this RCT. Global improvement was assessed by the Patient Global Impression of Improvement Scale (PGII) [41] and the Clinical Global Impression of Improvement Scale (CGII) [38].

### Risk of bias of individual studies

**Fig. 2:**
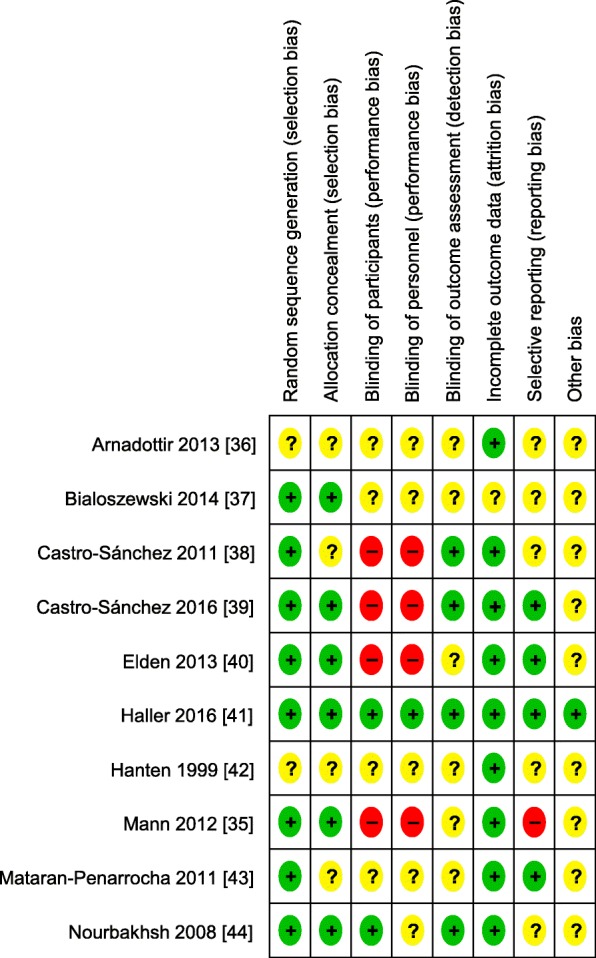
Risk of bias of individual studies

**Fig. 3:**
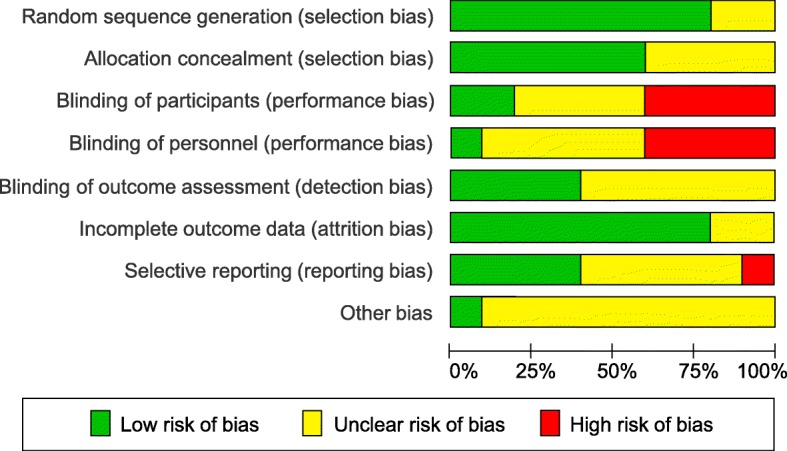
Risk of bias summary

The risk of bias assessments are summarized in Figs. 2 and 3. Risk of selection bias was assessed as low in 60% of the included studies [35, 37, 39–41, 44]. Two further trials reported adequate random sequence generation but did not provide information about allocation concealment [38, 43]. Two trials [41, 44] ensured blinding of participants. However, the overall risk of performance bias was unclear or high for all but one of the trials, as the therapists could not be blinded to treatment allocation or this information was missing. We assessed one of the RCTs [41] as having low risk of performance bias, although the therapists were not described as being blinded, as secondary analyses had shown that the quality of the alliance to the assigned therapists did not systematically affect study outcomes [45]. Adequate blinding of outcome assessors was reported by 40% of the studies [38, 39, 41, 44], whereas 60% did not provide sufficient information to assess the risk of detection bias. The risk of attrition bias was evaluated as low in 90% of the studies [35, 36, 38–44], the risk of selective reporting as low in 40% [39–41, 43]. The risk of other bias was assessed as unclear in 90% of the RCTs because of missing alpha-level adjustment [35, 38–40] or information about sources of funding [36, 37, 42–44]. The other studies reported having received no funding [38, 39], university funding [41], or government research grants [35, 40]. One of the trials also reported partial funding from CST associations for the publication fee [41].

### Risk of publication bias

Although funnel plots could not be created, the risk of publication bias is likely to be low. Searches of trial registries and conference proceedings revealed only one unpublished study [35], which could be included, as the trial authors provided all relevant data upon request. Manual searches of non-peer reviewed literature revealed two further RCTs [32, 33]. One of these [32] only reported rates of response for those whose quality of life improved but did not define improvement, the other RCT [33] did not report the sample sizes for each study group. By dividing the total N by 2, the calculated between-group effect sizes appeared unexpectedly high in favor of CST. Thus, the exclusion of those two trials will most probably not raise the risk of publication bias.

### Assessment of overall effect sizes

#### Effects on primary outcomes

**Fig. 4:**
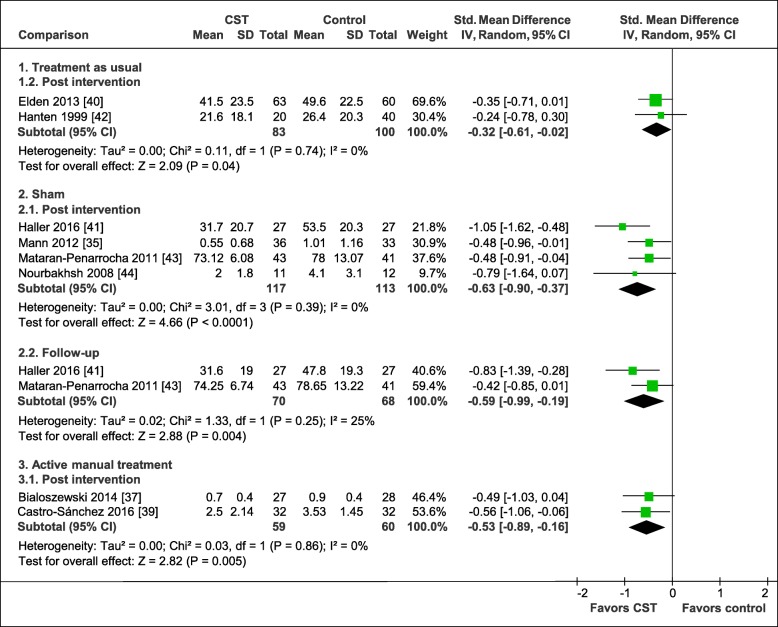
Forest plot of pain intensity

The pooled effects on pain intensity are shown in Fig. 4. In comparison to treatment as usual, CST showed a significant greater effect of a small size directly after the intervention (2 RCTs, SMD = − 0.32, 95%CI = [− 0.61, − 0.02], I^2^ = 0%, *N* = 183) [40, 42]. In comparison to manual and non-manual sham treatments, CST showed a significant medium pooled effect directly post intervention (4 RCTs, SMD = − 0.63, 95%CI = [− 0.90, − 0.37], I^2^ = 0%, *N* = 230) [35, 41, 43, 44]. By analyzing manual [41, 44] and non-manual sham controls [35, 43] separately, CST was found to be superior to manual sham with a greater pooled effect size (2 RCTs, SMD = − 0.97, 95%CI = [− 1.44, − 0.49], I^2^ = 0%, *N* = 77) compared to non-manual sham (2 R CTs, SMD = − 0.48, 95%CI = [− 0.80, − 0.16], I^2^ = 0%, *N* = 153). At 6-months follow-up, the pooling of the effects resulted in a significant medium effect size in favor of CST (2 RCTs, SMD = − 0.59, 95%CI = [− 0.99, − 0.19], I^2^ = 25%, *N* = 138) [41, 43] in comparison to manual and non-manual sham. In comparison to an active manual control treatment directly after the intervention, CST was found to produce greater effects resulting in a significant medium pooled effect size (2 RCTs, SMD = − 0.53, 95%CI = [− 0.89, − 0.16], I^2^ = 0%, *N* = 119) [37, 39].

**Fig. 5:**
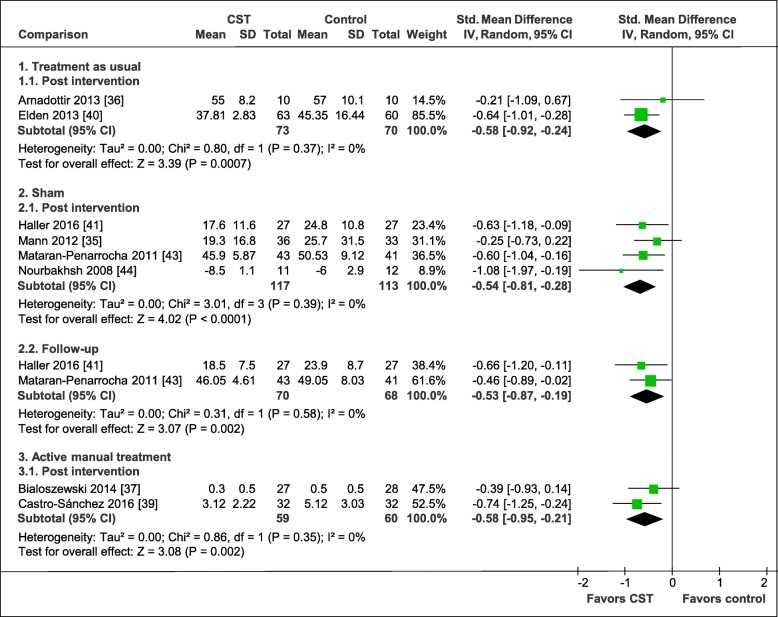
Forest plot of functional disability

The pooled effects on functional disability are shown in Fig. 5. In comparison to treatment as usual post intervention, the pooling of effects resulted in a significant greater medium effect size in favor of CST (2 RCTs, SMD = − 0.58, 95%CI = [− 0.92, − 0.24], I^2^ = 0%, *N* = 143) [36, 40]. In comparison to manual and non-manual sham, the meta-analysis showed a medium post-intervention effect (4 RCTs, SMD = − 0.54, 95%CI = [− 0.81, − 0.28], I^2^ = 0%, *N* = 230) [35, 41, 43, 44], while the separate pooling of RCTs testing CST against manual sham controls were found to have a greater effect (2 RCTs, SMD = − 0.76, 95%CI = [− 1.22, − 0.29], I^2^ = 0%, *N* = 77) [41, 44] than RCTs testing CST against non-manual sham controls (2 RCTs, SMD = − 0.44, 95%CI = [− 0.78, − 0.10], I^2^ = 10%, *N* = 153) [35, 43]. The meta-analysis at 6-months resulted in a significant medium effect size in favor of CST (2 RCTs, SMD = − 0.53, 95%CI = [− 0.87, − 0.19], I^2^ = 0%, *N* = 138) [41, 43] in comparison to manual and non-manual sham. For the comparison to an active manual control treatment post intervention, the pooling of the study data revealed a significant greater effect of a medium size in favor of CST (2 RCTs, SMD = − 0.58, 95%CI = [− 0.95, − 0.21], I^2^ = 0%, *N* = 119) [37, 39].

#### Effects on secondary outcomes

**Fig. 6:**
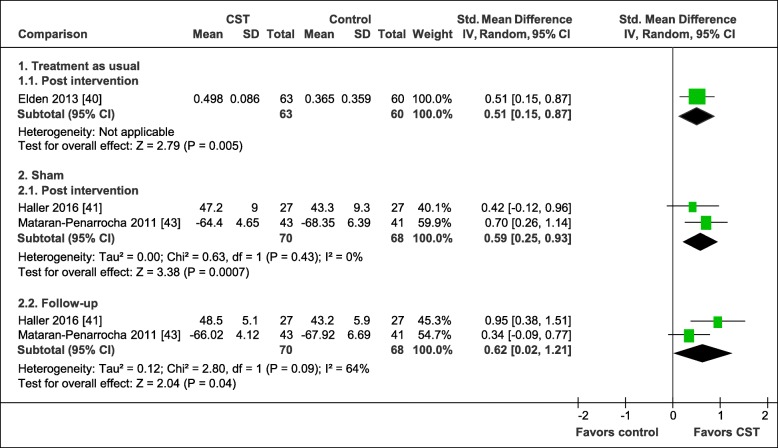
Forest plot of physical quality of life

The pooled effects on physical quality of life are shown in Fig. 6. In comparison to treatment as usual, the performed analysis revealed a significant greater medium post-intervention effect in favor of CST (1 RCT, SMD = 0.51, 95%CI = [0.15, 0.87], *N* = 123) [40]. In comparison to manual and non-manual sham conditions, the meta-analyses revealed a significant medium post-intervention effect (2 RCTs, SMD = 0.59, 95%CI = [0.25, 0.93], I^2^ = 0%, *N* = 138) [41, 43] as well as a significant medium 6-months follow-up effect (2 RCTs, SMD = 0.62, 95%CI = [0.02, 1.21], I^2^ = 64%, *N* = 138) [41, 43] in favor of CST. However, the meta-analysis of the follow-up effects revealed significant heterogeneity.

**Fig. 7:**
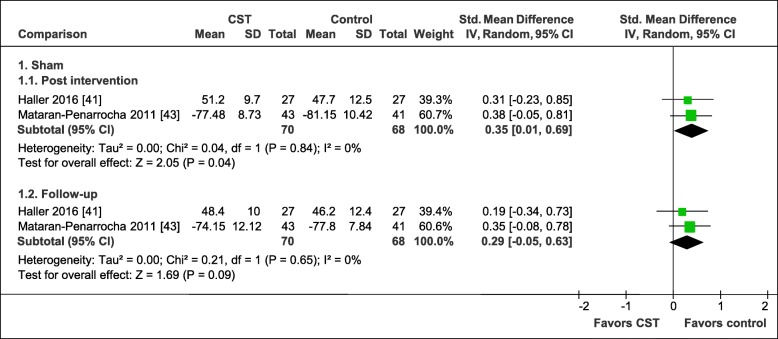
Forest plot of mental quality of life

The pooled effects on mental quality of life are shown in Fig. 7. In comparison to manual and non-manual sham controls, the meta-analyses revealed small pooled effects in favor of CST, which were found to be significant directly after the intervention (2 RCTs, SMD = 0.35, 95%CI = [0.01, 0.69], I^2^ = 0%, *N* = 138) [41, 43] but no longer at the 6-month follow-up (2 RCTs, SMD = 0.29, 95%CI = [− 0.05, 0.63], I^2^ = 0%, *N* = 138) [41, 43].

**Fig. 8:**
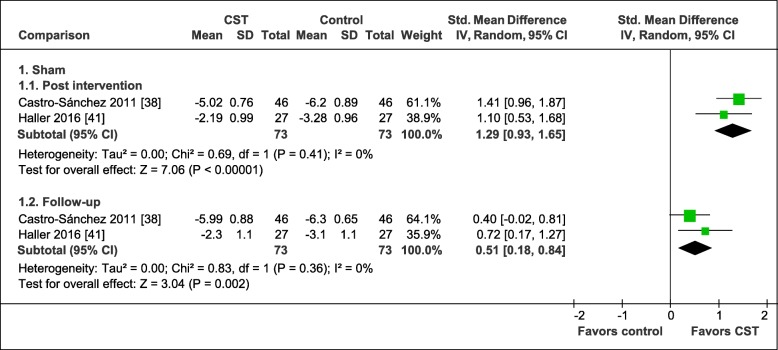
Forest plot of global improvement

The pooled effects on global improvement are shown in Fig. 8. In comparison to manual and non-manual sham controls, the meta-analyses resulted in a significant large pooled effect in favor of CST post intervention (2 RCTs, SMD = 1.29, 95%CI = [0.93, 1.65], I^2^ = 0%, *N* = 146) [38, 41] and a significant medium pooled effect six months after randomization (2 RCTs, SMD = 0.51, 95%CI = [0.18, 0.84], I^2^ = 0%, *N* = 146) [38, 41].

### Sensitivity analyses

By excluding studies with an unclear or high risk of the respective bias from the comparisons to treatment as usual, CST effects on pain intensity post intervention were found to be robust only against the risk of attrition bias, while the effects on functional disability and physical quality of life were found to be robust against the risk of selection, attrition, and reporting bias.

In comparison to manual and non-manual sham treatments, CST effects on pain intensity and functional disability post intervention as well as six months after randomization were still found to be significant even if the respective studies with unclear or high risk of selection, performance, detection, attrition, reporting, and other source of bias were excluded. The effects of CST on physical and mental quality of life in comparison to sham post intervention were found to be robust only against the risk of attrition and reporting bias. The significant follow-up effect on physical quality of life were robust against all risk of bias dimensions. This is also true for the post intervention and follow-up analyses on global improvement.

Sensitivity analyses of the comparisons to active manual controls revealed robust CST effects on pain intensity and functional disability post intervention against the risk of selection, detection, attrition, and reporting bias.

However, most of the sensitivity analyses included only one remaining RCT that had a low risk of the respective bias, with the exception of the analyses of pain intensity and functional disability in comparison to sham post intervention. These meta-analyses included always 2 to 3 of the 4 initially analyzed RCTs. Detailed analyses can be found in the Additional file 1.

### Safety

Five RCTs [35–37, 42, 44] provided no information about AEs. Two RCTs stated no withdrawal due to AEs [38, 39], while another reported no AEs at all [43]. The two remaining RCTs found 5 and 7 minor AEs in the CST group in comparison to 6 and 9 minor AEs in the groups receiving treatment as usual [40] and manual sham [41]. Minor AEs during or subsequent to the CST treatment included increased intensity of pain, headache, shivering, drowsiness, tiredness, and strong emotional reactions such as weeping. No serious adverse events were reported [40, 41].

## Discussion

### Summary of evidence

The systematic search revealed 10 RCTs investigating the efficacy and effectiveness of CST in pain patients with different chronic diagnoses. In comparison to treatment as usual, this meta-analysis found significant small to medium size pooled effects of CST directly after the end of the intervention for: pain intensity, functional disability, and physical quality of life, which was however based mainly on one RCT in patients with pelvic girdle pain. The effects on pain intensity were not robust against all, but one risk of bias domain; those on functional disability and physical quality were not robust against the risk of performance, detection and other bias. In comparison to manual and non-manual sham controls, CST resulted in significantly greater pooled effects of a medium to large size directly after the end of the intervention as well as six months after randomization for pain intensity, functional disability, physical quality of life, and global improvement. Effects tended to be higher in comparisons of studies with blinded patients as well as patients with neck pain or lateral epicondylitis compared to those with fibromyalgia or migraine. Six months after randomization, mental quality of life was no longer found to be significantly different to sham. All analyses towards sham were robust against all risk of bias domains, except for the effects of CST on physical and mental quality of life post intervention that were found to be robust only against the risk of attrition and reporting bias. In comparison to another active manual control, post-intervention data were available for meta-analysis of the effects on pain intensity and functional disability in patients suffering from low back pain. Both comparisons revealed significantly greater medium effect sizes in favor of CST and were robust against the risk of selection, detection, attrition, and reporting bias. No serious AEs were reported. Minor AEs were equally distributed between the groups, while patients receiving CST tended to report less AEs than those randomized to the treatment as usual or manual sham group. In general, however, the included RCTs did not sufficiently report adverse events.

### Advances on prior systematic reviews

In comparison to prior systematic reviews that included observational studies and RCTs [20], mixed cranial osteopathic and CST techniques [22], and healthy and clinical participants [24], this analysis focused on RCTs investigating CST in patients with chronic pain diagnoses. By searching published as well as unpublished studies, we were able to include one additional RCT that showed less positive results and was missed by the previous reviews [35]. Thus, we performed the first meta-analysis of CST trials that revealed no statistical heterogeneity except for one follow-up analysis; although it contained some clinical heterogeneity regarding the length of the CST interventions and the pain diagnoses of the patients. A further important issue for research and clinical practice are safety analyses that are not part of many previous reviews of CST [22, 24, 46].

### Limitations of the review

The first limitation is the small number of studies included in the meta-analysis. Conclusions drawn, especially those from analyses that included only 2 RCTs, remain preliminary [26]. We used Hedges’ correction for small samples and found no statistical heterogeneity in almost all meta-analyses, but adding a few more studies may change the significance of the results. Additionally, the large effect on global improvement may be overestimated, as retrospective data tend to be more vulnerable to recall biases [47]. A second limitation is the often unclear risk of bias profile of the included RCTs. Many RCTs did not report allocation concealment, blinding of outcome assessment, and alternative methods of decreasing the risk of performance bias. Risk of bias assessment may be influenced by the fact that four of the review authors (HH, RL, GD, and HC) conducted one of the included RCTs. However, a fifth review author (TS) independently assessed the risk of bias of the RCTs. As a result, we do not substantially deviate from the risk of bias assessment performed by previous reviews [20, 22]. The third limitation is a lack of subgroup analyses. We were neither able to derive conclusions about CST efficacy or effectiveness for special pain diagnoses, nor for the requisite number of treatment sessions. The fourth limitation is the focus on patient-reported outcomes, which are more vulnerable to the risk of detection bias. A fact that reduces the reliability of specific CST effects [22]. However, two of the included RCTs [41, 44] blinded patients to group allocation effectively; and three [39, 41, 44] used additional objective measures of pain and function, which in part led to short-term effects comparable to those assessed with self-reported measures. Another point that argues against specific CST effects is the mostly unclear or high risk of performance bias due to the lack of blinding therapists to group allocation. This issue was only controlled within one sham-controlled RCT [41] that showed that the quality of therapeutic alliance, rated by the blinded patients, did not significantly influence patient-reported outcomes [45]. Additionally, the light-touch sham control group used was credible to patients. Analyses confirmed that both expectation and credibility ratings were no significant predictors of group allocation [45]. In contrast, other control groups used in the analyzed RCTs did not achieve comparable levels of expectation or credibility compared to CST [48]. Besides, results gained from waiting list and comparative effectiveness trials should be interpreted with restraint as none of the RCTs controlled for patient expectations. Thus, placebo effects on pain cannot be excluded, although they were calculated as ranging just between a SMD of − 0.35 and − 0.16 [49].

### Implications for further research

Further clinical trials on CST are required. Authors should ensure rigorous methodology and reporting [50] as well as adequate controls for nonspecific therapy and therapist effects in order to reduce the risk of performance and detection bias. Even though therapists could not be blinded, controlling for attention effects by e.g. asking the patients about their perception of the therapeutic alliance [51] would be feasible. In waiting list or comparative effectiveness trials, where patients could not be blinded, patients’ expectations should be operationalized as a covariate and included in statistical analyses. In general, more adequate statistics (including intention-to-treat analyses as well as alpha-level adjustment for multiple testing) would ensure a low risk of attrition bias and other sources of bias. Increased attention should also be drawn to the adequate assessment and reporting of AEs and reasons for drop-out.

### Implications for clinical practice

The summarized evidence suggests robust short-term efficacy and comparative effectiveness of CST on pain intensity and functional disability. Longer-term effects seem plausible as well. According to this meta-analysis, CST was not associated with serious adverse events. However, clinicians should be aware of the potential risks of forcibly applied spinal CST techniques, which ca be associated with serious AEs, particularly in patients with preexisting pathologies of the spine [52]. Nonetheless, CST seem to be as safe as other conventional or commentary manual treatments [52] and might provide a novel treatment option in cases where standard treatments have failed to cause symptom alleviation. Recommendations for specific pain conditions cannot be given.

## Conclusions

This meta-analysis suggests significant and robust effects of CST on pain and function, which are not exclusively explainable by placebo responses or effects due to non-specific treatment mechanisms. More RCTs strictly following CONSORT are needed to further corroborate the efficacy, comparative effectiveness, and safety of CST in patients with chronic pain conditions.

## Supplementary information

**Additional file 1.** Sensitivity analyses.

## Data Availability

The datasets used and/or analyzed during the current study are available from the corresponding author on reasonable request.

## Abbreviations

AE: Adverse Event
CI: Confidence Interval
CONSORT: Consolidated Standards of Reporting Trials
CST: Craniosacral Therapy
I^2^: Statistical Heterogeneity
N: Sample Size
PRISMA: Preferred Reporting Items for Systematic Reviews and Meta-Analyses
RCT: Randomized Controlled Trial
SD: Standard Deviation
SMD: Standard Mean Difference
