# Supplementary material for: Craniosacral therapy for chronic pain: a systematic review and meta-analysis of randomized controlled trials
Source: BMC Musculoskelet Disord. 2019 Dec 31;21:1. doi: 10.1186/s12891-019-3017-y (PMC6937867; doi:10.1186/s12891-019-3017-y)
Supplement: Supplementary file 1 — Additional file 1. Sensitivity analyses. [file 12891_2019_3017_MOESM1_ESM.pdf]

Supplementary material to: Craniosacral Therapy for chronic pain: a systematic review and meta-analysis of randomized controlled trials

By: Heidemarie Haller, Romy Lauche, Tobias Sundberg, Gustav Dobos, and Holger Cramer

Sensitivity analysis including only trails with low risk of selection bias:

Comparison	Outcome	Time point	Result	Change in significance
CST versus TAU	Pain intensity	Post intervention	1/2 RCTs, SMD = -0.35, 95%CI = [-0.71, 0.01], N = 123	Yes
	Functional disability	Post intervention	1/2 RCTs, SMD = -0.64, 95%CI = [-1.01, -0.28], N = 123	No
	Physical quality of life	Post intervention	1/1 RCT, SMD = 0.51, 95%CI = [0.15, 0.87], N = 123	No
CST versus SHAM	Pain intensity	Post intervention	3/4 RCTs, SMD = -0.73, 95%CI = [-1.09, -0.38], I^2 = 10%, N = 146	No
		6-month follow-up	1/2 RCTs, SMD = -0.83, 95%CI = [-1.39, -0.28], N = 54	No
	Functional disability	Post intervention	3/4 RCTs, SMD = -0.55, 95%CI = [-0.96, -0.13], I^2 = 31%, N = 146	No
		6-month follow-up	1/2 RCTs, SMD = -0.66, 95%CI = [-1.20, -0.11], N = 54	No
	Physical quality of life	Post intervention	1/2 RCTs, SMD = 0.42, 95%CI = [-0.12, 0.96], N = 54	Yes
		6-month follow-up	1/2 RCTs, SMD = 0.95, 95%CI = [0.38, 1.51], N = 54	No
	Mental quality of life	Post intervention	1/2 RCTs, SMD = 0.31, 95%CI = [-0.23, 0.85], N = 54	Yes
		6-month follow-up	No significant overall effects.	––
	Global improvement	Post intervention	1/2 RCTs, SMD = 1.10, 95%CI = [0.53, 1.68], N = 54	No
		6-month follow-up	1/2 RCTs, SMD = 0.72, 95%CI = [0.17, 1.27], N = 54	No
CST versus AMT	Pain intensity	Post intervention	2/2 RCTs, SMD = -0.53, 95%CI = [-0.89, -0.16], I^2 = 0%, N = 119	No
	Functional disability	Post intervention	2/2 RCTs, SMD = -0.58, 95%CI = [-0.95, -0.21], I^2 = 0%, N = 119	No

Sensitivity analysis including only trails with low risk of performance bias:

Comparison	Outcome	Time point	Result	Change in significance
CST versus TAU	Pain intensity	Post intervention	The analysis did not contain trails with low risk of performance bias.	––
	Functional disability	Post intervention	The analysis did not contain trails with low risk of performance bias.	––
	Physical quality of life	Post intervention	The analysis did not contain trails with low risk of performance bias.	––
CST versus SHAM	Pain intensity	Post intervention	1/4 RCTs, SMD = -1.05, 95%CI = [-1.62, -0.48], N = 54	No
		6-month follow-up	1/2 RCTs, SMD = -0.83, 95%CI = [-1.39, -0.28], N = 54	No
	Functional disability	Post intervention	1/4 RCTs, SMD = -0.63, 95%CI = [-1.18, -0.09], N = 54	No
		6-month follow-up	1/2 RCTs, SMD = -0.66, 95%CI = [-1.20, -0.11], N = 54	No
	Physical quality of life	Post intervention	1/2 RCTs, SMD = 0.42, 95%CI = [-0.12, 0.96], N = 54	Yes
		6-month follow-up	1/2 RCTs, SMD = 0.95, 95%CI = [0.38, 1.51], N = 54	No
	Mental quality of life	Post intervention	1/2 RCTs, SMD = 0.31, 95%CI = [-0.23, 0.85], N = 54	Yes
		6-month follow-up	No significant overall effects.	––
	Global improvement	Post intervention	1/2 RCTs, SMD = 1.10, 95%CI = [0.53, 1.68], N = 54	No
		6-month follow-up	1/2 RCTs, SMD = 0.72, 95%CI = [0.17, 1.27], N = 54	No
CST versus AMT	Pain intensity	Post intervention	The analysis did not contain trails with low risk of performance bias.	––
	Functional disability	Post intervention	The analysis did not contain trails with low risk of performance bias.	––

Sensitivity analysis including only trails with low risk of detection bias:

Comparison	Outcome	Time point	Result	Change in significance
CST versus TAU	Pain intensity	Post intervention	The analysis did not contain trails with low risk of detection bias.	–
	Functional disability	Post intervention	The analysis did not contain trails with low risk of detection bias.	–
	Physical quality of life	Post intervention	The analysis did not contain trails with low risk of detection bias.	–
CST versus SHAM	Pain intensity	Post intervention	2/4 RCTs, SMD = -0.97, 95%CI = [-1.44, -0.49], I^2 = 0%, N = 77	No
		6-month follow-up	1/2 RCTs, SMD = -0.83, 95%CI = [-1.39, -0.28], N = 54	No
	Functional disability	Post intervention	2/4 RCTs, SMD = -0.76, 95%CI = [-1.22, -0.29], I^2 = 0%, N = 77	No
		6-month follow-up	1/2 RCTs, SMD = -0.66, 95%CI = [-1.20, -0.11], N = 54	No
	Physical quality of life	Post intervention	1/2 RCTs, SMD = 0.42, 95%CI = [-0.12, 0.96], N = 54	Yes
		6-month follow-up	1/2 RCTs, SMD = 0.95, 95%CI = [0.38, 1.51], N = 54	No
	Mental quality of life	Post intervention	1/2 RCTs, SMD = 0.31, 95%CI = [-0.23, 0.85], N = 54	Yes
		6-month follow-up	No significant overall effects.	–
	Global improvement	Post intervention	2/2 RCTs, SMD = 1.29, 95%CI = [0.93, 1.65], I^2 = 0%, N = 146	No
		6-month follow-up	2/2 RCTs, SMD = 0.51, 95%CI = [0.18, 0.84], I^2 = 0%, N = 146	No
CST versus AMT	Pain intensity	Post intervention	1/2 RCTs, SMD = -0.56, 95%CI = [-1.06, -0.06], N = 64	No
	Functional disability	Post intervention	1/2 RCTs, SMD = -0.74, 95%CI = [-1.25, -0.24], N = 64	No

Sensitivity analysis including only trails with low risk of attrition bias:

Comparison	Outcome	Time point	Result	Change in significance
CST versus TAU	Pain intensity	Post intervention	2/2 RCTs, SMD = -0.32, 95%CI = [-0.61, -0.02], I^2 = 0%, N = 183	No
	Functional disability	Post intervention	2/2 RCTs, SMD = -0.58, 95%CI = [-0.92, -0.24], I^2 = 0%, N = 143	No
	Physical quality of life	Post intervention	1/1 RCT, SMD = 0.51, 95%CI = [0.15, 0.87], N = 123	No
CST versus SHAM	Pain intensity	Post intervention	4/4 RCTs, SMD = -0.63, 95%CI = [-0.90, -0.37], I^2 = 0%, N = 230	No
		6-month follow-up	2/2 RCTs, SMD = -0.59, 95%CI = [-0.99, -0.19], I^2 = 25%, N = 138	No
	Functional disability	Post intervention	4/4 RCTs, SMD = -0.54, 95%CI = [-0.81, -0.28], I^2 = 0%, N = 230	No
		6-month follow-up	2/2 RCTs, SMD = -0.53, 95%CI = [-0.87, -0.19], I^2 = 0%, N = 138	No
	Physical quality of life	Post intervention	2/2 RCTs, SMD = 0.59, 95%CI = [0.25, 0.93], I^2 = 0%, N = 138	No
		6-month follow-up	2/2 RCTs, SMD = 0.62, 95%CI = [0.02, 1.21], I^2 = 64%, N = 138	No
	Mental quality of life	Post intervention	2/2 RCTs, SMD = 0.35, 95%CI = [0.01, 0.69], I^2 = 0%, N = 138	No
		6-month follow-up	No significant overall effects.	–
	Global improvement	Post intervention	2/2 RCTs, SMD = 1.29, 95%CI = [0.93, 1.65], I^2 = 0%, N = 146	No
		6-month follow-up	2/2 RCTs, SMD = 0.51, 95%CI = [0.18, 0.84], I^2 = 0%, N = 146	No
CST versus AMT	Pain intensity	Post intervention	1/2 RCTs, SMD = -0.56, 95%CI = [-1.06, -0.06], N = 64	No
	Functional disability	Post intervention	1/2 RCTs, SMD = -0.74, 95%CI = [-1.25, -0.24], N = 64	No

Sensitivity analysis including only trails with low risk of reporting bias:

**Comparison**	**Outcome**	**Time point**	**Result**	**Change in significance**
CST versus TAU	Pain intensity	Post intervention	1/2 RCTs, SMD = -0.35, 95%CI = [-0.71, 0.01], N = 123	Yes
	Functional disability	Post intervention	1/2 RCTs, SMD = -0.64, 95%CI = [-1.01, -0.28], N = 123	No
	Physical quality of life	Post intervention	1/1 RCT, SMD = 0.51, 95%CI = [0.15, 0.87], N = 123	No
CST versus SHAM	Pain intensity	Post intervention	2/4 RCTs, SMD = -0.73, 95%CI = [-1.29, -0.18], I^2 = 59%, N = 138	No
		6-month follow-up	1/2 RCTs, SMD = -0.83, 95%CI = [-1.39, -0.28], N = 54	No
	Functional disability	Post intervention	2/4 RCTs, SMD = -0.61, 95%CI = [-0.96, -0.27], I^2 = 0%, N = 138	No
		6-month follow-up	2/2 RCTs, SMD = -0.53, 95%CI = [-0.87, -0.19], I^2 = 0%, N = 138	No
	Physical quality of life	Post intervention	2/2 RCTs, SMD = 0.59, 95%CI = [0.25, 0.93], I^2 = 0%, N = 138	No
		6-month follow-up	2/2 RCTs, SMD = 0.62, 95%CI = [0.02, 1.21], I^2 = 64%, N = 138	No
	Mental quality of life	Post intervention	2/2 RCTs, SMD = 0.35, 95%CI = [0.01, 0.69], I^2 = 0%, N = 138	No
		6-month follow-up	No significant overall effects.	–
	Global improvement	Post intervention	1/2 RCTs, SMD = 1.10, 95%CI = [0.53, 1.68], N = 54	No
		6-month follow-up	1/2 RCTs, SMD = 0.72, 95%CI = [0.17, 1.27], N = 54	No
CST versus AMT	Pain intensity	Post intervention	1/2 RCTs, SMD = -0.56, 95%CI = [-1.06, -0.06], N = 64	No
	Functional disability	Post intervention	1/2 RCTs, SMD = -0.74, 95%CI = [-1.25, -0.24], N = 64	No

Sensitivity analysis including only trails with low risk of other bias:

**Comparison**	**Outcome**	**Time point**	**Result**	**Change in significance**
CST versus TAU	Pain intensity	Post intervention	The analysis did not contain trails with low risk of other bias.	–
	Functional disability	Post intervention	The analysis did not contain trails with low risk of other bias.	–
	Physical quality of life	Post intervention	The analysis did not contain trails with low risk of other bias.	–
CST versus SHAM	Pain intensity	Post intervention	1/4 RCTs, SMD = -1.05, 95%CI = [-1.62, -0.48], N = 54	No
		6-month follow-up	1/2 RCTs, SMD = -0.83, 95%CI = [-1.39, -0.28], N = 54	No
	Functional disability	Post intervention	1/4 RCTs, SMD = -0.63, 95%CI = [-1.18, -0.09], N = 54	No
		6-month follow-up	1/2 RCTs, SMD = -0.66, 95%CI = [-1.20, -0.11], N = 54	No
	Physical quality of life	Post intervention	1/2 RCTs, SMD = 0.42, 95%CI = [-0.12, 0.96], N = 54	Yes
		6-month follow-up	1/2 RCTs, SMD = 0.95, 95%CI = [0.38, 1.51], N = 54	No
	Mental quality of life	Post intervention	1/2 RCTs, SMD = 0.31, 95%CI = [-0.23, 0.85], N = 54	Yes
		6-month follow-up	No significant overall effects.	–
	Global improvement	Post intervention	1/2 RCTs, SMD = 1.10, 95%CI = [0.53, 1.68], N = 54	No
		6-month follow-up	1/2 RCTs, SMD = 0.72, 95%CI = [0.17, 1.27], N = 54	No
CST versus AMT	Pain intensity	Post intervention	The analysis did not contain trails with low risk of other bias.	–
	Functional disability	Post intervention	The analysis did not contain trails with low risk of other bias.	–
